# Dipeptidyl Peptidase‐4 Inhibitor Use and Risk of Pemphigus Vulgaris and Foliaceus: A Retrospective Cohort Study Using Databases of the Korea National Health Insurance Claims and the National Health Screening Examination

**DOI:** 10.1111/1346-8138.70419

**Published:** 2026-07-22

**Authors:** Jaein Lee, Jae Hyun Bae, Seokyung Hahn

**Affiliations:** ^1^ Interdisciplinary Program of Medical Informatics Seoul National University College of Medicine Seoul South Korea; ^2^ Doctors Dermatology Clinic Seoul South Korea; ^3^ Division of Endocrinology and Metabolism, Department of Internal Medicine Seoul National University Hospital Seoul South Korea; ^4^ Department of Internal Medicine Seoul National University College of Medicine Seoul South Korea; ^5^ Department of Human Systems Medicine Seoul National University College of Medicine Seoul South Korea; ^6^ Medical Bigdata Research Center, Medical Research Center Seoul National University Seoul South Korea; ^7^ Institute of Health Policy and Management, Medical Research Center Seoul National University Seoul Korea

**Keywords:** clone‐censor‐weight method, dipeptidyl peptidase 4 inhibitors, extended cox proportional hazard model, pemphigus foliaceus, pemphigus vulgaris

## Abstract

Although population‐based studies have reported an increased risk of bullous pemphigoid and pemphigus associated with dipeptidyl peptidase‐4 (DPP‐4) inhibitors, there is limited, well‐designed epidemiological evidence for pemphigus vulgaris (PV), which has only been supported by case reports thus far. This study aimed to assess whether DPP‐4 inhibitor use in type 2 diabetes patients is associated with PV and pemphigus foliaceus (PF) development. A retrospective cohort study was conducted using the Korean National Health Insurance databases. The study included 1 228 526 patients newly diagnosed with type 2 diabetes over a period of 11 years. Hazard ratios (HRs) were calculated using an extended Cox proportional hazards model, target trial approach with clone‐censor‐weight (CCW) method, and inverse‐probability‐of‐treatment weighting (IPTW) analysis. We found that DPP‐4 inhibitor use was associated with increased PV risk (HR: 3.22, 95% CI: 1.03–10.04 [Cox]; HR: 2.24, 95% CI: 1.04–4.84 [IPTW]; HR: 1.69, 95% CI: 0.63–4.51 [CCW]). The association was not observed with PF risk (HR: 0.89, 95% CI: 0.25–3.14 [Cox]; HR: 0.97, 95% CI: 0.39–2.42 [CCW]; and HR: 0.56, 95% CI: 0.23–1.41 [IPTW]). These findings suggest that DPP‐4 inhibitors may not only induce bullous pemphigoid, but also severe autoimmune blistering diseases such as pemphigus vulgaris. Clinicians should recognize the possibility of such severe adverse effects when evaluating new‐onset bullous lesions in patients taking DPP‐4 inhibitors.

## 1 Introduction

Pemphigus is a rare acquired autoimmune bullous disease that can affect the skin and mucous membranes. If left untreated, it can be life‐threatening [1]. Pemphigus vulgaris (PV) is the most common subtype, with an annual incidence rate ranging from 0.1 to 0.5 per 100 000 people [2], depending on the region and ethnicity. Another prevalent subtype is pemphigus foliaceus (PF), which differs in its immunopathological features [3]. PV is caused by autoantibodies against desmoglein 3 (Dsg3) and/or desmoglein 1 (Dsg1), whereas PF involves antibodies against Dsg1 [4]. These autoantibodies bind to desmosomes in keratinocytes, leading to acantholysis and blister formation.

Multiple epidemiologic studies, including case–control studies [5], cohort studies [6, 7, 8, 9, 10], and systematic reviews [11] have established that dipeptidyl peptidase‐4 (DPP‐4) inhibitors increase bullous pemphigoid (BP) risk through upregulation of proinflammatory cytokines, particularly eotaxin, which activates cutaneous eosinophils [12].

While a recent nationwide population‐based study in Taiwan reported an increased risk of pemphigus as an aggregate entity, epidemiological evidence focusing specifically on the PV and PF remains limited [13]. To date, case reports have documented PV occurrence in DPP‐4 inhibitor users, potentially via increased tumor necrosis factor‐α (TNF‐α), interleukin‐1 (IL‐1), and interleukin‐6 (IL‐6), but this association lacks epidemiological validation [14, 15]. Furthermore, while DPP‐4 inhibitors may theoretically increase PF risk through similar cytokine dysregulation, this potential link has not yet been systematically investigated.

Given DPP‐4 inhibitors' widespread use in type 2 diabetes and the serious morbidity of PV and PF, we conducted the first large‐scale cohort study examining the association between DPP‐4 inhibitor use and PV/PF using multiple analytic approaches in a nationwide population.

## 2 Methods

### 2.1 Data Sources, Study Design, and Patient Selection

A retrospective cohort was established using the Korean National Health Insurance claims database and National Health Screening Program (NHSP) examination database. The cohort included type 2 diabetes patients (ICD‐10‐CM diagnosed 2002–2020) with first‐ever antidiabetic prescriptions. We excluded those with prior diabetes (2002–2009), age ≤ 30 years, or no NHSP examination within 2 years of diagnosis. Additional exclusions included HIV infection, prior autoimmune bullous disorders [16], type 1 diabetes, and insulin as first‐line therapy, as they represent a group with different disease severity (Figure S1) [6]. The Institutional Review Board of Seoul National University waived informed consent for this anonymized retrospective study (Approval No. E‐2206‐075‐1332). Detailed information on data sources, study design, and patient selection criteria is provided in Appendix S1.

### 2.2 Study Outcomes and Follow‐Up

The outcome of the study was the development of PV or PF. Cases were identified using International Classification of Diseases (ICD)‐10‐CM codes (L10.0 and L10.2, respectively) together with rare, intractable disease registration codes (V132 and V210, respectively) from the Korean NHI system. These V codes, which provide up to 90% coverage of medical expenses, are assigned exclusively to patients who meet stringent diagnostic criteria. These criteria include histopathologic evidence of intraepidermal blistering with acantholysis, as well as either (1) direct immunofluorescence demonstrating epidermal immunoglobulin G deposition or (2) serologic detection of autoantibodies to Dsg 1 or 3 by indirect immunofluorescence or enzyme‐linked immunosorbent assay (ELISA). The follow‐up period was defined as the time from the commencement of continuous diabetes medication administration without interruption until the earliest of the following events: the occurrence of PV (for PV analyses) or PF (for PF analyses), the end of continuous diabetes medication administration, or the end of the study period (December 31, 2020). The term “continuous administration of a diabetes medication” was defined as uninterrupted medication use, covering the period from the start of the initial prescription through to the last day of the last prescription. The term “extension period” was defined as the allowed gap between the end of a prescription period and the subsequent visit. During this period, it was hypothesized that the patient was taking the medication continuously. The default extension period was set at 30 days. The administration of continuous diabetes medication is terminated under two circumstances: first, when the patient fails to attend the scheduled clinic visit within the allowed extension period; and second, when the patient dies. For patients without a history of PV or PF events, the outcome data were censored at the end of continuous administration of a diabetes medication or the end of the study period.

### 2.3 Exposure Definition

Diabetes medication prescriptions for patients with type 2 diabetes can change over time and can be divided into two mutually exclusive categories. The exposure group comprised individuals receiving DPP‐4 inhibitors either as monotherapy or in combination with other diabetes drugs. The comparator group included those treated with non–DPP‐4 inhibitor diabetes medications (non‐DPP4i‐DMRx; metformin, sulfonylureas, glucagon‐like peptide 1 [GLP‐1] receptor agonists, sodium‐glucose cotransporter 2 inhibitors, meglitinides, thiazolidinediones, alpha‐glucosidase inhibitors used as monotherapy, combination therapy, or switching to insulin or adding on insulin). Study participants could switch between DPP4 inhibitors and non‐DPP4i‐DMRx during follow‐up.

### 2.4 Covariates

The covariates included age at the time of the initial prescription for diabetes medication, sex, comorbidities, and NHSP variables. The presence of comorbidities was determined by referring to data from a minimum of 3 years prior to the registration date of the cohort, using the operational definitions provided in Table S1. These comorbidities included psoriasis [17], dementia [18], Parkinson's disease [18], epilepsy [18], and any cancer [19], all of which have been reported to be associated with pemphigus. The NHSP variables included body mass index (BMI), waist circumference, systolic blood pressure, diastolic blood pressure, and fasting blood glucose.

### 2.5 Reporting

The results were reported according to the Strengthening the Reporting of Observational Studies in Epidemiology (STROBE) guidelines.

### 2.6 Statistical Analysis

Antidiabetic medication prescription patterns were classified into three base study groups (Figure 1). To address immortal time bias from the temporal discrepancy between cohort entry and DPP‐4 inhibitor initiation [20, 21], the primary analysis employed an extended Cox proportional hazards model (Model 1) treating diabetes medication as a time‐dependent variable. Model 1 was selected as the primary framework because it represents the most streamlined clinical scenario, accommodating exactly three utilization patterns: continuous non‐DPP4i‐DMRx use, continuous DPP‐4 inhibitor use, and a single, unidirectional transition from initial non‐DPP4i‐DMRx to DPP‐4 inhibitors without subsequent reversal. This framework is methodologically analogous to an irreversible, non‐surgical‐to‐surgical transition, providing an intuitive clinical benchmark.

**FIGURE 1:**
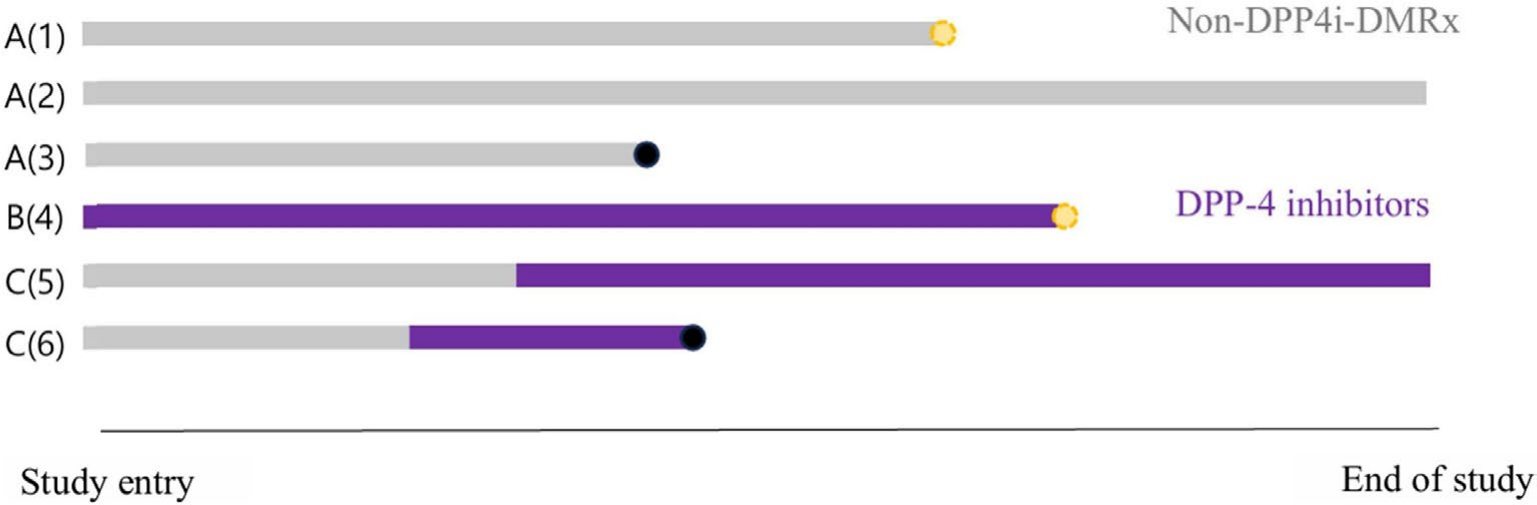
Definition of the event and survival time by prescribing patterns for time‐varying analysis (Model 1). All potential prescribing patterns of DPP‐4 inhibitors and non‐DPP‐4 inhibitor diabetes medications (non‐DPP4i‐DMRx) in type 2 diabetes were analyzed, considering a single continuous DPP‐4 inhibitor prescription for the occurrence of pemphigus vulgaris (PV) when analyzing the risk of PV, and as the occurrence of pemphigus foliaceus (PF) when analyzing the risk of PF. The study population can be divided into three categories: (A) The initial diabetes medication is non‐DPP4i‐DMRx, which is continued until the prescription regimen ends with the discontinuation of non‐DPP4i‐DMRx therapy (1–3), (B) The initial diabetes medication is a DPP‐4 inhibitor, which is continued until the prescription regimen ends with the discontinuation of DPP‐4 inhibitor therapy (4), (C) The initial use of non‐DPP4i‐DMRx is followed by a switch to a DPP‐4 inhibitor (5, 6). The black dots indicate the occurrence of PV when the risk of PV is analyzed, as well as the occurrence of PF when the risk of PF is analyzed. The orange dots indicate the loss of follow‐up.

To comprehensively evaluate our findings across alternative methodologies and varying causal assumptions, other modeling approaches were investigated. A target trial approach with the clone‐censor‐weight (CCW) method (Model 2) was applied to address a distinct clinical question focusing on the timing of treatment initiation within a predefined 2‐year grace period (Figure 2; Table S9). We conducted additional time‐dependent analyses to reflect greater real‐world treatment complexity that deviates from the unidirectional transition assumption. Model 3 was an extension of Model 1 that additionally accounted for a single reverse transition, including patients who initiated DPP‐4 inhibitors but later switched back to non–DPP4i‐DMRx, while Model 4 further extended this approach to allow multiple unrestricted switches between treatment groups throughout follow‐up. Schematics of these approaches are provided in Figures S2 and S3. We also performed an inverse‐probability‐of‐treatment weighting (IPTW) analysis (Model 5) using stabilized IPTW to balance covariates between groups. In the event of potential influence from extreme weights, we conducted a sensitivity analysis using Winsorization to restrict the estimated weights. This process replaced values outside the first and 99th percentiles with their respective thresholds instead of excluding the observations [22]. Table S2 describes the five statistical approaches. We additionally examined pemphigus risk according to individual DPP‐4 inhibitors, constructing separate study populations for each drug using Model 1. Subgroup analyses evaluated potential effect modification by age (< 65 vs. ≥ 65 years), sex, and BMI. Additional analyses redefined the extension period in Model 1 from 30 to 60, 90, and 180 days. Models 1–4 were adjusted for baseline covariates described in the methods section, and robust variance estimation was applied in Models 3 and 4 to account for patients contributing multiple episodes. We conducted a sensitivity analysis using Firth's penalized likelihood method to reduce small‐sample bias and improve the stability of hazard estimates in the presence of few observed events [23]. Hazard ratios (HRs) and 95% confidence intervals (CIs) were estimated for all models. Statistical significance was defined as a two‐sided *p* < 0.05. Further methodological details for all models and additional analyses are provided in Appendix S2. Analyses were conducted using SAS Enterprise Guide 7.15 (SAS Institute, Cary, NC).

**FIGURE 2:**
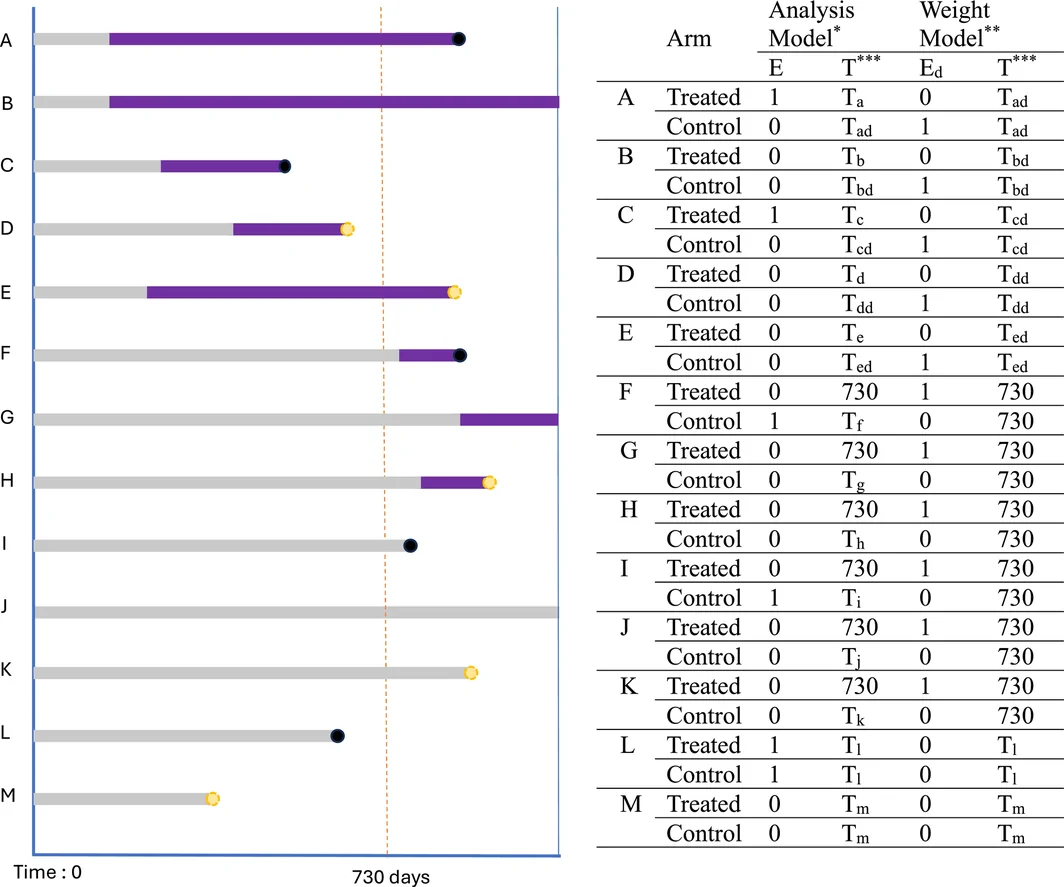
Definition and illustration of the outcome and survival time for each patient by each arm, as used in both the weight and analysis models. In the clone‐censor‐weight approach (Model 2), the definitions of patient‐specific outcomes and survival times for each arm used in the weighting and analysis models are as follows: Patients A–E have a history of DPP‐4 inhibitor use within the designated grace period (730 days), contributing to the weighting model until the initiation of a DPP‐4 inhibitor. In the weight model, the censoring metric is set to 1 for the control group, indicating that the patient is no longer within the protocol. In contrast, the metric is set to 0 for the treatment group, indicating that the patient remains within the protocol after initiating a DPP‐4 inhibitor during the grace period. For the analysis model in the control arm, these patients are classified as censored upon initiation of a DPP‐4 inhibitor. Patients F–K meet the criteria for the control group and contribute to the weighted model for the entire grace period (730 days). In the weight model for the control group, they have a censoring indicator of 0, while in the treatment group, they have a censoring indicator of 1. In the analysis model for the treated arm, these patients are censored at Day 730. Patients L and M contribute equally to both arms (analytic and weighted models) due to their compliance with the protocol. The black dots indicate the occurrence of PV when analyzing the risk of PV and the occurrence of PF when analyzing the risk of PF. Orange dots indicate that follow‐up was lost. *Event of interest: PV or PF occurrence at each analysis (E = 1); **event of interest: Deviation from protocol (E_d_ = 1); ***time in days used as follow‐up time in the analysis or weight model; T_X_: Time to PV or PF occurrence at each analysis or time to censoring for patient X; T_Xd_: Time to DPP‐4 inhibitors for patient X; Treated = Patients treated with DPP‐4 inhibitors (purple); Control = Patients treated with non‐DPP‐4 inhibitor diabetes medications (gray).

## 3 Results

From January 1, 2010, to December 31, 2020, 2 536 268 individuals were newly diagnosed with type 2 diabetes. After applying exclusion criteria, 1 228 526 individuals comprised the final cohort (Figure S4). The baseline characteristics of patients who initiated DPP‐4 inhibitors versus non‐DPP4i‐DMRx are presented in Table S3. In the cohort, 74.0% of patients did not switch between DPP‐4 inhibitors and non‐DPP4i‐DMRx during follow‐up; 16.9% switched once, 4.8% switched twice, 2.5% switched three times, and 1.9% switched four times or more. None of the cases diagnosed as either pemphigus vulgaris or foliaceus were found to be bullous pemphigoid.

### 3.1 Pemphigus Vulgaris

Time‐varying analyses for Models 1, 3, and 4 revealed that patients who had used a DPP‐4 inhibitor had a higher risk of developing PV than patients who had used non‐DPP4i‐DMRx. The hazard ratios (HRs) for the use of a DPP‐4 inhibitor were 3.22 (95% confidence interval [CI]: 1.03–10.04), 3.31 (95% CI: 1.06–10.32), and 3.35 (95% CI: 1.21–9.30), for Models 1, 3, and 4, respectively. The CCW approach (Model 2) revealed that the DPP‐4 inhibitor group had a non‐significantly increased risk of PV compared with the non‐DPP4i‐DMRx group (HR: 1.69, 95% CI: 0.63–4.51). The IPTW analysis (Model 5) revealed that the DPP‐4 inhibitor group had a significantly increased risk of PV compared with the non‐DPP4i‐DMRx group (HR: 2.24, 95% CI: 1.04–4.84) (Table 1).

**TABLE 1:** Results of the association between the use of DPP‐4 inhibitors and the risk of pemphigus vulgaris and pemphigus foliaceus using various statistical methods.

Exposure	Outcome	No. of patients	No. of events	Person‐years	Incidence rate (per 100 000 py)	Crude HR (95% CI)	Adjusted HR (95% CI)
Time‐varying analysis (Model 1)	PV						
Non‐DPP4i‐DMRx		900 814	5	1 092 047	0.46	1.00 (Reference)	1.00 (Reference)
DPP‐4 inhibitors		573 192	8	895 086	0.89	3.09 (1.01–9.48)	3.22 (1.03–10.04)
CCW method (Model 2)	PV						
Non‐DPP4i‐DMRx		1 227 992	5.7^a^ (5^b^)	1604941^a^	0.36^a^		1.00 (Reference)
DPP‐4 inhibitors		1 227 992	13^a^ (13^b^)	1430962^a^	0.91^a^		1.69 (0.63–4.51)
Time‐varying analysis (Model 3)	PV						
Non‐DPP4i‐DMRx		974 830	5	1 158 321	0.43	1.00 (Reference)	1.00 (Reference)
DPP‐4 inhibitors		573 192	8	893 995	0.89	3.18 (1.04–9.76)	3.31 (1.06–10.32)
Time‐varying analysis (Model 4)	PV						
Non‐DPP4i‐DMRx		974 830	6	1 259 849	0.48	1.00 (Reference)	1.00 (Reference)
DPP‐4 inhibitors		573 192	11	1 041 475	1.06	3.08 (1.12–8.46)	3.35 (1.21–9.30)
IPTW (Model 5)	PV						
Non‐DPP4i‐DMRx		653 438	10^a^ (5^b^)	1480103^a^	0.69^a^		1.00 (Reference)
DPP‐4 inhibitors		571 304	18^a^ (8^b^)	1890645^a^	0.95^a^		2.24 (1.04–4.84)
Time‐varying analysis (Model 1)	PF						
Non‐DPP4i‐DMRx		900 814	7	1 092 035	0.64	1.00 (Reference)	1.00 (Reference)
DPP‐4 inhibitors		573 192	4	895 093	0.45	0.84 (0.24–2.88)	0.89 (0.25–3.14)
CCW method (Model 2)	PF						
Non‐DPP4i‐DMRx		1 227 992	8.5^a^ (7^b^)	1604925^a^	0.53^a^		1.00 (Reference)
DPP‐4 inhibitors		1 227 992	10^c^ (10^b^)	1430968^a^	0.70^a^		0.97 (0.39–2.43)
Time‐varying analysis (Model 3)	PF						
Non‐DPP4i‐DMRx		974 829	8	1 158 306	0.69	1.00 (Reference)	1.00 (Reference)
DPP‐4 inhibitors		573 192	4	894 002	0.45	0.77 (0.23–2.56)	0.83 (0.24–2.84)
Time‐varying analysis (Model 4)	PF						
Non‐DPP4i‐DMRx		974 829	8	1 259 836	0.64	1.00 (Reference)	1.00 (Reference)
DPP‐4 inhibitors		573 192	4	1 041 486	0.38	0.74 (0.22–2.48)	0.80 (0.23–2.73)
IPTW (Model 5)	PF						
Non‐DPP4i‐DMRx		653 438	12^a^ (7^b^)	1480092^a^	0.83^a^		1.00 (Reference)
DPP‐4 inhibitors		571 304	7^a^ (4^b^)	1890663^a^	0.40^a^		0.56 (0.23–1.41)

*Note:* Model 1: time‐varying analysis using an extended Cox regression model; Model 2: clone‐censor‐weight (CCW) approach; Model 3: time‐varying analysis allowing switching to non‐DPP4i‐DMRx after using a DPP‐4 inhibitor as the first diabetes treatment; Model 4: time‐varying analysis allowing switching between the use of DPP‐4 inhibitors and non‐DPP4i‐DMRx on multiple occasions (to simulate real‐world clinical practice); Model 5: inverse‐probability‐of‐treatment weighting (IPTW) analysis using the stabilized IPTW between the DPP‐4 inhibitor and non‐DPP4i‐DMRx groups (used to minimize confounding by indication). Refer to Table S2 in the supplement for a more detailed explanation of the different models.

Abbreviations: CCW, clone‐censor‐weight; CI, confidence interval; DPP‐4, dipeptidyl peptidase‐4; HR, hazard ratio; IPTW, inverse probability‐of‐treatment weighting; non‐DPP4i‐DMRx, non‐DPP‐4 inhibitor diabetes medications; PF, pemphigus foliaceus; PV, pemphigus vulgaris; py, person‐years.

^a^ These are weighted values.

^b^ These are unweighted values.

^c^ This is a weighted value, rounded to 10 from the actual value of 10.00000023.

The individual DPP‐4 inhibitor analysis revealed that patients who had used linagliptin had a markedly elevated risk of developing PV, compared with patients who had used non‐DPP4i‐DMRx (adjusted HR: 6.05, 95% CI: 1.42–25.80). However, the Likelihood Ratio Test for the overall model fit did not reach significance (*p* = 0.80). While sitagliptin, alogliptin, and teneligliptin were found to have hazard ratios greater than one, these associations did not reach statistical significance. The risks associated with vildagliptin, saxagliptin, gemigliptin, anagliptin, and evogliptin use could not be evaluated owing to the absence of events (Table 2).

**TABLE 2:** Analysis of the association between the use of individual DPP‐4 inhibitors and the risk of PV using time‐varying analysis.

Exposure					
Individual drug	PV events	Person‐years	Incidence rate (per 100 000 person‐years)	Crude HR (95% CI)	Adjusted HR (95% CI)
Non‐DPP4i‐DMrx	4	1 092 615	0.37	1.00 (Reference)	1.00 (Reference)
Sitagliptin	2	215 636	0.93	3.58 (0.65–19.65)	4.18 (0.74–23.48)
Non‐DPP4i‐DMrx	6	1 092 744	0.55	1.00 (Reference)	1.00 (Reference)
Vildagliptin	0	60 879	0	NA	NA
Non‐DPP4i‐DMrx	5	1 092 781	0.46	1.00 (Reference)	1.00 (Reference)
Saxagliptin	0	35 120	0	NA	NA
Non‐DPP4i‐DMrx	5	1 092 562	0.46	1.00 (Reference)	1.00 (Reference)
Linagliptin	3	168 208	1.78	5.59 (1.33–3.48)	6.05 (1.42–25.80)^a^
Non‐DPP4i‐DMrx	5	1 092 659	0.46	1.00 (Reference)	1.00 (Reference)
Gemigliptin	0	114 660	0	NA	NA
Non‐DPP4i‐DMrx	5	1 092 735	0.46	1.00 (Reference)	1.00 (Reference)
Alogliptin	1	35 128	2.85	7.11 (0.83–60.91)	7.69 (0.88–67.65)
Non‐DPP4i‐DMrx	5	1 092 726	0.46	1.00 (Reference)	1.00 (Reference)
Teneligliptin	1	42 903	2.33	5.97 (0.70–51.21)	6.24 (0.71–54.57)
Non‐DPP4i‐DMrx	5	1 092 776	0.46	1.00 (Reference)	1.00 (Reference)
Anagliptin	0	13 151	0	NA	NA
Non‐DPP4i‐DMrx	5	1 092 755	0.46	1.00 (Reference)	1.00 (Reference)
Evogliptin	0	19 977	0	NA	NA

Abbreviations: CI, confidence interval; DPP‐4, dipeptidyl peptidase‐4; HR, hazard ratio; NA, not applicable; non‐DPP4i‐DMRx, non‐DPP‐4 inhibitor diabetes medications.

^a^ Testing global null hypothesis, *p* = 0.298.

In the time‐varying analysis (Model 1), patients who had used a DPP‐4 inhibitor were at a higher risk of developing PV than patients using non‐DPP4i‐DMRx when the extension period was increased to 60 days (HR: 2.91, 95% CI: 1.02–8.25; *p* = 0.07 for the Likelihood Ratio Test for model fit) (Table S4). By increasing the extension period to 90 and 180 days, a higher risk of PV in patients using DPP‐4 inhibitors was observed with marginal significance (HR: 2.55, 95% CI: 0.94–6.89; and HR: 2.52, 95% CI: 0.98–6.45, respectively) (Table S4). Any potential interactions by age, sex, and BMI were not detected (Table S5). Firth's penalized Cox regression showed a consistent association (HR 3.07; 95% CI 1.05–9.76). A sensitivity analysis using weights winsorized at the 1st and 99th percentiles to mitigate the influence of extreme weights in Model 5 yielded an adjusted HR of 2.19 (95% CI, 1.01–4.43). The detailed weight distributions for Model 5 before and after Winsorization are presented in Table S10.

### 3.2 Pemphigus Foliaceus

None of the time‐varying model analyses (Models 1, 3, and 4) captured a significant association between DPP‐4 inhibitor use and PF development (HR: 0.89, 95% CI: 0.25–3.14; HR: 0.83, 95% CI: 0.24–2.84; and HR: 0.80, 95% CI: 0.23–2.73, respectively). Similar non‐significant associations were observed in the CCW approach (Model 2) and IPTW analysis (model 5) (HR, 0.97; 95% CI, 0.39–2.42 and HR, 0.56; 95% CI, 0.23–1.41, respectively) (Table 1). Despite elevated HRs, none of the individual DPP‐4 inhibitors demonstrated a statistically significant association with PF development: teneligliptin (HR, 5.37; 95% CI, 0.63–45.42), sitagliptin (HR, 0.98; 95% CI, 0.12–8.29), and linagliptin (HR, 1.48; 95% CI, 0.17–12.65). The risk of PF associated with vildagliptin, saxagliptin, gemigliptin, alogliptin, anagliptin, and evogliptin use could not be evaluated because no PF events were observed (Table S6). With increased extension periods of 60, 90, or 180 days, PF risk did not differ significantly (Table S7). Subgroup analyses by age, sex, and BMI showed no significant interactions (Table S8).

## 4 Discussion

Several systematic reviews link DPP‐4 inhibitors to BP [11, 24, 25, 26, 27], but evidence for PV is limited to case reports although several hypotheses have been proposed on the potential association between DPP‐4 inhibitors and PV. One hypothesis is that DPP‐4 inhibitors may alter immune responses and skin inflammation [28]. The DPP‐4 enzyme, also known as the CD26 cell surface antigen, is present in a variety of tissues, including T lymphocytes, and endothelial and epithelial cells [29, 30]. CD26 is expressed at high levels on CD4+ T cells, which are associated with the production of T helper (Th1) cytokines, including interferon‐gamma and interleukin‐2 [30]. A Th1‐cell deficiency has been associated with an increased risk of developing PV [31]. Another hypothesis involves disrupted degradation of desmoglein 1 (Dsg1) by kallikrein‐related peptidase 5, influenced by DPP‐4 inhibitors altering DPP‐4 and KLK5 activity, leading to enhanced autoantibody targeting of Dsg1 [14, 32, 33, 34]. DPP‐4 inhibitors may also increase proinflammatory cytokines (TNF‐α, IL‐1β, IL‐6, CXCL12) [14, 15, 35, 36], facilitating tertiary lymphoid structures and autoantibody‐producing B cells in skin lesions. In contrast, GLP‐1 receptor agonists, another incretin therapy, lack these immunologic actions and are not linked to PV. Notably, the selective association of DPP‐4 inhibitors with PV but not pemphigus foliaceus (PF), despite both involving Dsg1 autoantibodies, suggests DPP‐4 inhibitors may specifically impact immune responses involving Dsg3. Further research is needed to clarify these mechanisms.

A prior pharmacovigilance study reported an association between DPP‐4 inhibitors and pemphigus [37]. Consistent with this, we demonstrated a significant association between DPP‐4 inhibitors, particularly linagliptin, and PV, though not with sitagliptin or alogliptin. This discrepancy likely reflects variations in data sources and diagnostic accuracy. While the previous study used a self‐reported database without subtype differentiation, our analysis focused on subtype‐specific, biopsy‐proven events. Of the DPP‐4 inhibitors, only linagliptin was associated with a significantly higher hazard ratio for PV when compared to non‐DPP4i‐DMRx. Linagliptin has the longest half‐life of all DPP‐4 inhibitors (approximately 200 h [38]), potentially prolonging these immune dysregulation mechanisms. Thus, the half‐life of DPP‐4 inhibitors may influence the risk of developing PV. Nevertheless, these drug‐specific findings warrant cautious interpretation. Prioritizing diagnostic precision severely limited our statistical power when evaluating individual agents. This limitation may have obscured true associations for other medications, especially more recently introduced agents like gemigliptin, alogliptin, anagliptin, and evogliptin, where the absence of observed events is likely due to short follow‐up and limited clinical exposure rather than a definitive lack of risk.

DPP‐4 inhibitor use was associated with PV at the 30‐day extension period but not at 60 days or beyond, contrasting with BP, which shows sustained elevated risk with longer extension periods [6]. This differential pattern suggests distinct pathophysiological mechanisms between PV and BP. Although BP incidence is higher in older adults and men, effect modification by these factors has not been clearly established [5, 9]. In our study, PV and PF incidence did not significantly vary by age or sex, with no evidence of effect modification.

We assessed the consistency of the findings using an extended Cox proportional hazards model, CCW method, and IPTW analyses. Overall, the direction of the association between DPP‐4 inhibitor use and PV risk was generally consistent across models, although the magnitude and statistical significance of the HR estimates differed according to the exposure definition and analytic framework. Models 1, 3, and 4, which dynamically allocated person‐time according to treatment exposure throughout follow‐up, consistently suggested an increased PV risk associated with DPP‐4 inhibitor use while minimizing immortal time bias. In contrast, Model 2 (CCW method) showed a lower and non‐significant HR estimate, likely reflecting differences in treatment classification within the predefined 2‐year grace period and the limited statistical power resulting from the extreme rarity of PV. Specifically, patients who initiated DPP‐4 inhibitors after the grace period were classified into the control group under the CCW framework, which may have attenuated the estimated HR. Similarly, the lower HR observed in the IPTW analysis may reflect residual immortal time bias related to exposure classification. Despite these quantitative differences, the overall upward trend in HR estimates across models supports the possibility of an association between DPP‐4 inhibitor use and increased PV risk, with Model 1 providing the most representative estimate of real‐world clinical practice by dynamically capturing treatment exposure throughout the entire follow‐up period.

We developed three different models (Models 1, 3, and 4) using time‐varying covariates for DPP‐4 inhibitor use. These models were designed to evaluate the consistency of the findings under an alternative medication scenario that may occur in clinical practice. The observed association remained consistent even after accounting for treatment transitions between DPP‐4 inhibitors and non‐DPP4i‐DMRx. Although additional treatment transitions resulted in additional follow‐up time in Model 3, no additional PV events were observed, likely reflecting the extreme rarity of PV. Given the low incidence rate of PV (approximately 0.43 per 100 000 person‐years in the Model 3), the additional follow‐up time contributed by patients who switched treatment did not yield more PV events. Nevertheless, the switching framework remained operational, as reflected by changes observed in PF events. Therefore, Model 3 serves as a consistency check for our primary findings rather than an independent replication.

As previously noted, the rarity of PV inherently resulted in a limited number of events and reduced statistical power. A post hoc power calculation based on Hsieh and Lavori's method [39] indicated an estimated statistical power of approximately 53.8%. Although this level is below the conventional threshold for a definitive confirmatory analysis, the detection of a significant association despite this limited power suggests that the observed association may warrant further investigation as a potentially noteworthy biological signal. To address potential bias related to the low events‐per‐variable ratio, we performed a sensitivity analysis using Firth's penalized likelihood Cox regression [23]. The consistency between the penalized estimate (HR 3.07; 95% CI 1.05–9.76) and our primary analysis supports the findings and suggests that the observed association is unlikely to be explained solely by the low events‐per‐variable ratio. Furthermore, the use of a longitudinal cohort design with time‐varying covariates, rather than a static case–control approach, enabled assessment of the temporal relationship between DPP‐4 inhibitor exposure and the development of PV throughout follow‐up. However, given the wide confidence intervals and limited number of events, these findings should be considered hypothesis‐generating.

To assess the robustness of the weighted analyses, we evaluated weight stability using the effective sample size (ESS) and performed sensitivity analyses. Model 5 exhibited instability due to extreme weights in the non‐DPP4i‐DMRx group, with a maximum weight of 1126.57, which reduced the ESS to 50.0% of the original sample size. After applying the Winsorization method [22], the ESS recovered to 94.9%, and the adjusted HR remained consistent (HR 2.19; 95% CI 1.01–4.43), suggesting that the findings were not driven by extreme weights. In contrast, the CCW method showed stable weights without additional adjustment. Nevertheless, the unweighted number of PV events remained below 20 across all models. Therefore, although weighting improved covariate balance and weight stability, it could not compensate for the limited number of events.

A recent nationwide, population‐based cohort study in Taiwan reported an elevated risk of pemphigus as an aggregate entity [13]. While the Taiwanese study evaluated pemphigus as a broad category, our analysis separately assessed pemphigus subtypes and identified a significant association with PV but not PF. This distinction may be relevant from a pathophysiological perspective, given the different antigenic targets and autoantibody profiles among pemphigus subtypes, and may help guide future mechanistic studies. Furthermore, our study applied a more stringent case definition by requiring both ICD‐10 coding and registration in the South Korean rare disease registry (V‐codes), which requires definitive diagnostic confirmation. Although this approach reduced the number of events, it likely minimized outcome misclassification.

While our findings consistently suggested an association between DPP‐4 inhibitor use and PV risk across multiple analytical approaches, the underlying biological mechanisms could not be evaluated directly in this study. Owing to the nature of population‐based epidemiological studies using large‐scale healthcare databases, mechanistic evidence was beyond the scope of the present investigation. Therefore, we relied on existing biological hypotheses from bullous pemphigoid studies and case reports of DPP‐4 inhibitor‐induced pemphigus to interpret the observed association.

To our knowledge, this is the first cohort study to investigate the association between DPP‐4 inhibitors and PV using a large nationwide health insurance database linked with a rare disease registry (V codes), which ensures histopathologic and immunological confirmation. In addition, integration with the National Health Screening Examination database enabled adjustment for clinical biometric variables, including fasting glucose, BMI, waist circumference, and systolic blood pressure, providing more comprehensive control of metabolic confounding than claims‐only datasets. In addition, our study was based on a new‐onset type 2 diabetes cohort, allowing assessment of DPP‐4 inhibitor exposure from an earlier stage of diabetes management and more precise control for diabetes severity and metabolic status. Further considerations are also required for interpretation of our study results. First, DPP‐4 inhibitor monotherapy and combination therapy were analyzed together, potentially obscuring individual drug efficacy. Second, the residual impact of DPP‐4 inhibitors in the control group during time‐varying analysis may have underestimated hazard ratios, though the association remained significant. Finally, it should be acknowledged that the limited number of events and wide confidence intervals remain important limitations; therefore, our findings should be interpreted as hypothesis‐generating rather than confirmatory. Nevertheless, the present study provides complementary evidence and further validation through large‐scale multinational registry‐based studies is warranted.

In conclusion, PV showed an increased association with DPP‐4 inhibitor use, suggesting a possible drug‐related relationship. DPP‐4 inhibitors may induce not only BP but also severe autoimmune blistering diseases such as PV. Clinicians should recognize this serious adverse effect and promptly evaluate new‐onset bullous lesions in patients receiving DPP‐4 inhibitors. However, this finding requires confirmation in other cohorts.

## Funding

This work was supported by the National Research Foundation of Korea (Grant Nos.: 2022R1A2C101043711 and RS‐2025‐00559766).

## Ethics Statement

The Institutional Review Board of Seoul National University waived informed consent for this anonymized retrospective study (Approval No. E‐2206‐075‐1332).

## Conflicts of Interest

The authors declare no conflicts of interest.

## Supporting information


**Figure S1:** Washout and enrollment in the study.
**Figure S2:** Time‐varying analysis (Model 3).
**Figure S3:** Time‐varying analysis (Model 4).
**Figure S4:** Flow diagram of cohort selection.
**Table S1:** Operational definition of disease.
**Table S2:** Summary of various statistical methods.
**Table S3:** Baseline characteristics of patients with new‐onset type 2 diabetes mellitus who underwent health screening 2 years preceding the date of diabetes diagnosis in the original data and after inverse probability of treatment weighting.
**Table S4:** Analysis of the association between DPP‐4 inhibitor use and the risk of pemphigus vulgaris (PV) using time‐varying analysis over different extension periods, including the 60‐, 90‐, and 180‐day extension periods.
**Table S5:** Subgroup analysis of the association between DPP‐4 inhibitor use and the risk of pemphigus vulgaris (PV) using a time‐varying analysis.
**Table S6:** Analysis of the association between the use of individual DPP‐4 inhibitors and the risk of pemphigus foliaceus (PF) using time‐varying analysis.
**Table S7:** Analysis of the association between DPP‐4 inhibitor use and the risk of pemphigus foliaceus (PF) using time‐varying analysis over different extension periods, including the 60‐, 90‐, and 180‐day extension periods.
**Table S8:** Subgroup analysis of the association between DPP‐4 inhibitor use and pemphigus foliaceus (PF) risk using a time‐varying analysis.
**Table S9:** The specification of the target trial and its emulation.
**Table S10:** Distributions of inverse probability of treatment weights and effective sample sizes before and after Winsorization in the pemphigus vulgaris (PV) analysis.
**Appendix S1:** Details on the data sources, study design, and patient selection.
**Appendix S2:** Details on the statistical analysis.

## Data Availability

The data supporting this study are available from the Korean National Health Insurance databases, but they are not publicly accessible due to privacy regulations. Access to the data requires prior approval from the Korean National Health Insurance and is granted only after a formal review process. Approved researchers may access the data in a secure, designated environment provided by the Korean National Health Insurance in Myeong‐dong, Seoul. Accessing the data online or remotely is strictly prohibited. Researchers interested in accessing the data may contact author Jaein Lee for assistance with the application process.
