# Supplementary material for: Dipeptidyl Peptidase‐4 Inhibitor Use and Risk of Pemphigus Vulgaris and Foliaceus: A Retrospective Cohort Study Using Databases of the Korea National Health Insurance Claims and the National Health Screening Examination
Source: J Dermatol. 2026 Jul 22;53(10):1429–38. doi: 10.1111/1346-8138.70419 (PMC13646859; doi:10.1111/1346-8138.70419)
Supplement: Supplementary file 1 [file JDE-53-1429-s001.docx]

Dipeptidyl Peptidase-4 Inhibitor Use and Risk of Pemphigus Vulgaris and Foliaceus: A Retrospective Cohort Study using databases of the Korea National Health Insurance claims and the National Health Screening Examination

Supplementary files

Supplementary Figures

Supplementary Figure S1. Washout and enrollment in the study

Supplementary Figure S2. Time-varying analysis (Model 3)

Supplementary Figure S3. Time-varying analysis (Model 4)

Supplementary Figure S4. Flow diagram of cohort selection

Supplementary Tables

Supplementary Table S1. Operational definition of disease

Supplementary Table S2. Summary of various statistical methods

Supplementary Table S3. Baseline characteristics of patients with new-onset type 2 diabetes mellitus who underwent health screening 2 years preceding the date of diabetes diagnosis in the original data and after inverse probability of treatment weighting

Supplementary Table S4. Analysis of the association between DPP-4 inhibitor use and the risk of pemphigus vulgaris (PV) using time-varying analysis over different extension periods, including the 60-day, 90-day, and 180-day extension periods

Supplementary Table S5. Subgroup analysis of the association between DPP-4 inhibitor use and the risk of pemphigus vulgaris (PV) using a time-varying analysis

Supplementary Table S6. Analysis of the association between the use of individual DPP-4 inhibitors and the risk of pemphigus foliaceus (PF) using time-varying analysis

Supplementary Table S7. Analysis of the association between DPP-4 inhibitor use and the risk of pemphigus foliaceus (PF) using time-varying analysis over different extension periods, including the 60-day, 90-day, and 180-day extension periods

Supplementary Table S8. Subgroup analysis of the association between DPP-4 inhibitor use and pemphigus foliaceus (PF) risk using a time-varying analysis

Supplementary Table S9. The specification of the target trial and its emulation

Supplementary Table S10. Distributions of inverse probability of treatment weights and effective sample sizes before and after Winsorization in the pemphigus vulgaris (PV) analysis

Supplementary Appendix 1. Details on the data sources, study design, and patient selection

Supplementary Appendix 2. Details on the statistical analysis

-3y

Follow up period

-2y

Wash out period

2002.01.01

2020.12.31

2010.01.01 Start of study

Type 2 diabetes diagnosis

Health exam

0 (Time)

Comorbidities

Type 2 diabetes

prescription

(Enrollment)

**Supplementary Figure S1.** Washout and enrollment in the study

Study entry

End of study

DPP-4 inhibitors

A(1)

Non-DPP4i-DMRx

A(3)

C(6)

D(7)

D(8)

A(2)

B(4)

C(5)

**Supplementary Figure S2.** Time-varying analysis (Model 3)

All potential prescription patterns of DPP-4 inhibitors and non-DPP-4 inhibitor diabetes medications (non-DPP4i-DMRx) for type 2 diabetes regarding the occurrence of pemphigus vulgaris (PV) and pemphigus foliaceus (PF) were analyzed, considering a single continuous DPP-4 inhibitor prescription. The population can be divided into four categories, as follows: A) The initial diabetes medication is a non-DPP4i-DMRx, which is continued until the prescription regimen ends with the discontinuation of the non-DPP4i-DMRx (1, 2, 3). B) The initial diabetes medication is a DPP-4 inhibitor, which is continued until the prescription regimen ends with the discontinuation of DPP-4 inhibitor therapy (4).

C) The initial use of a non-DPP4i-DMRx is followed by a switch to a DPP-4 inhibitor (5, 6). D) The initial prescription is a DPP-4 inhibitor, which is subsequently switched to a non-DPP4i-DMRx (7,8). The black dots indicate the occurrence of PV when analyzing the risk of PV, or the occurrence of PF when analyzing the risk of PF, while the orange dots indicate the loss of follow-up.

Study entry

End of study

DPP-4 inhibitors

1

3

6

7

8

2

4

5

Non-DPP4i-DMRx

**Supplementary Figure S3.** Time-varying analysis (Model 4)

A comprehensive analysis of the prescribing patterns of DPP-4 inhibitors and non-DPP-4 inhibitor diabetes medications (non-DPP4i-DMRx) for type 2 diabetes, allowing for multiple switching between DPP-4 inhibitors and non-DPP4i-DMRx for the occurrence of pemphigus vulgaris (PV) when analyzing the risk of PV, and the occurrence of pemphigus foliaceus (PF) when analyzing the risk of PF. The black dots indicate the occurrence of PV when analyzing the risk of PV or the occurrence of PF when analyzing the risk of PF, while the orange dots indicate the loss of follow-up.

**Supplementary Figure S4.** Flow diagram of cohort selection

**Supplementary Table S1.** Operational definition of disease

| Disease | ICD-10-CM code | Rare incurable disease | Prescription or imaging test | Number of visits |
| --- | --- | --- | --- | --- |
| Type 2 diabetes | E11, E12, E13, E14 | N/A | Diabetes medication (initiate medication within one year of diagnosis) | At least one outpatient or inpatient visit |
| Pemphigus vulgaris | L10.0 | V132 | N/A | At least one outpatient or inpatient visit |
| Pemphigus foliaceus | L10.2 | V210 | N/A | At least one outpatient or inpatient visit |
| Dementia | F00, F01, F03, G30 | N/A | Acetylcholinesterase inhibitor | At least one outpatient or inpatient visit |
| Parkinson’s disease | G20 | V124 | N/A | At least one outpatient or inpatient visit |
| Epilepsy | G40, G41, F803, R56 | N/A | Anticonvulsant for at least 6 months | At least two outpatient visits |
| Cancer | C00-14, C15-26, C30-39, C40-41,C43-44, C45-49, C50, C51-58, C60-63, C64-68, C69-72, C73-75, C81-96 | V193 | N/A | At least one outpatient or inpatient visit |
| Psoriasis | L40 | N/A | Topical Vitamin D derivatives | At least one outpatient or inpatient visit |

N/A: Not applicable

**Supplementary Table S2.** Summary of various statistical methods

| Model | Statistical methods | Control | Treatment | Grace period | Study Groups |
| --- | --- | --- | --- | --- | --- |
| Time-varying analysis (Model 1) | Extended Cox proportional hazards model | Non-DPP4i-DMRx | DPP-4 inhibitors | - | Study Group A: Non-DPP4i-DMRx only users  Study Group B: DPP-4 inhibitors only users  Study Group C: Started with non-DPP4i-DMRx then switched to DPP-4 inhibitors |
| Clone-censor-weight method (Model 2) | Weighted Cox proportional hazards model | Non-DPP4i-DMRx | DPP-4 inhibitors within 2 years after diagnosis of type 2 diabetes | 2 years | Study Group A: Non-DPP4i-DMRx only users  Study Group B: DPP-4 inhibitors only users  Study Group C: Started with non-DPP4i-DMRx then switched to DPP-4 inhibitors  All individuals in the population were copied and assigned equally to the control and treatment groups at baseline. |
| Time-varying analysis (Model 3) | Extended Cox proportional hazards model | Non-DPP4i-DMRx | DPP-4 inhibitors | - | Study Group A: Non-DPP4i-DMRx only users  Study Group B: DPP-4 inhibitors only users  Study Group C: Started with non-DPP4i-DMRx then switched to DPP-4 inhibitors  Study Group D: Started with DPP-4 inhibitors then switched to Non-DPP4i-DMRx |
| Time-varying analysis (Model 4) | Extended Cox proportional hazards model | Non-DPP4i-DMRx | DPP-4 inhibitors | - | Multiple non-DPP4i-DMRx and DPP-4 inhibitors switching |
| Inverse probability of treatment weighting (Model 5) | Propensity scores were estimated using a logistic regression model, and outcomes were analyzed with a Cox proportional hazards model incorporating IPTW | Non-DPP4i-DMRx | DPP-4 inhibitors | - | Study Group A: Non-DPP4i-DMRx only users  Study Group B: DPP-4 inhibitors only users  Study Group C: Started with non-DPP4i-DMRx then switched to DPP-4 inhibitors  The population of individuals who used only non-DPP4i-DMRx was considered the control group. The population of individuals who used only DPP-4 inhibitors and those who used non-DPP4i-DMRx initially and then switched to DPP-4 inhibitors were regarded as the treatment group. |

DPP-4, dipeptidyl peptidase-4; non-DPP4i-DMRx, non-DPP-4 inhibitor diabetes medications. Extended Cox proportional hazards models were fitted using age at initial diabetes medication prescription, sex, comorbidities (psoriasis, dementia, Parkinson’s disease, epilepsy, and cancer), and National Health Screening Program measurements (body mass index, waist circumference, systolic/diastolic blood pressure, and fasting blood glucose) as covariates. Propensity scores were estimated via logistic regression with the same covariates

**Supplementary Table S3.** Baseline characteristics of patients with new-onset type 2 diabetes mellitus who underwent health screening 2 years preceding the date of diabetes diagnosis in the original data and after inverse probability of treatment weighting

|  | Original data | | |  | Inverse probability of treatment weighting data | | | | | |
| --- | --- | --- | --- | --- | --- | --- | --- | --- | --- | --- |
| Characteristics | DPP-4 inhibitors used  N = 571,304 | Non-DPP4i-DMRx  N = 653,438 | SMD | | |  | DPP-4 inhibitors used  N = 571,304 | Non-DPP4i-DMRx  N = 653,438 | SMD |  |
|  |  |  |  |  |  |  |  |  |  |  |
| Age, mean (SD), years | 55.5 (11.1) | 57.2 (11.4) | −0.14 | | |  | 56.3 (11.2) | 56.2 (11.5) | 0.01 |  |
| Male, n (%) | 356,379 (62.38%) | 386,965 (59.22%) | −0.06 | | |  | 348,266 (60.96%) | 401,145 (61.39%) | 0.01 |  |
| Waist Circumference,  mean (SD), cm | 87.81(9.43) | 87.36(9.72) | 0.05 | | |  | 87.64(9.27) | 87.63(10.85) | 0.001 |  |
| BMI, mean (SD), kg/m^2^ | 26.32(3.72) | 26.17(3.72) | 0.04 | | |  | 26.27(3.68) | 26.24(3.80) | 0.006 |  |
| SBP, mean (SD), mmHg | 129.79(15.67) | 129.56(15.59) | 0.01 | | |  | 129.70(15.59) | 129.71(15.67) | −0.001 |  |
| DBP, mean (SD), mmHg | 80.70(10.60) | 80.21(10.53) | 0.05 | | |  | 80.47(10.54) | 80.48(10.58) | −0.001 |  |
| FBS, mean (SD), mg/dL | 157.91(59.91) | 135.64(45.06) | 0.42 | | |  | 146.71(51.98) | 154.24(83.40) | −0.14 |  |
| Comorbidities, n (%) |  |  |  | | |  |  |  |  |  |
| Psoriasis | 3,599(0.63%) | 3,920(0.6%) | −0.004 | | |  | 3,542(0.62%) | 3,985(0.61%) | −0.001 |  |
| Dementia | 4,856(0.85%) | 7,841(1.2%) | 0.03 | | |  | 5,941(1.04%) | 6,730(1.03%) | −0.001 |  |
| Parkinson’s disease | 856(0.15% | 1,306(0.2%) | 0.01 | | |  | 1,028(0.18%) | 1,176(0.18%) | −0.001 |  |
| Epilepsy | 6,055(1.06%) | 7,645(1.17%) | 0.01 | | |  | 6,398(1.12%) | 7,253(1.11%) | −0.001 |  |
| Cancer | 27,879(4.88%) | 36,461(5.58%) | 0.03 | | |  | 29,993(5.25%) | 34,370(5.26%) | 0.001 |  |

BMI, body mass index; DBP, diastolic blood pressure; DPP-4, dipeptidyl peptidase-4; FBS, fasting blood sugar; non-DPP4i-DMRx, non-DPP-4 inhibitor diabetes medications; SBP, systolic blood pressure; SD: Standard deviation; SMD, standardized mean difference.

**Supplementary Table S4.** Analysis of the association between DPP-4 inhibitor use and the risk of pemphigus vulgaris (PV) using time-varying analysis over different extension periods, including the 60-day, 90-day, and 180-day extension periods.

| Exposure | Patients | Events  (PV) | Person-years | Incidence rate (per 100,000 person-years | Crude HR (95% CI) | Adjusted HR (95% CI) |
| --- | --- | --- | --- | --- | --- | --- |
| 60-day extension period |  |  |  |  |  |  |
| Non-DPP4i-DMRx | 900,852 | 6 | 1,311,448 | 0.46 | 1.00 (Reference) | 1.00 (Reference) |
| DPP-4 inhibitors | 613,921 | 10 | 1,115,009 | 0.90 | 2.79 (0.998–7.80) | 2.91* (1.02–8.25)* |
| 90-day extension period |  |  |  |  |  |  |
| NonDPP4i-DMRx | 900,873 | 7 | 1,407,905 | 0.50 | 1.00 (Reference) | 1.00 (Reference) |
| DPP-4 inhibitors | 633,368 | 10 | 1,213,387 | 0.82 | 2.41 (0.91–6.44) | 2.55 (0.94–6.89) |
| 180-day extension period |  |  |  |  |  |  |
| NonDPP4i-DMRx | 900,888 | 8 | 1,532,100 | 0.52 | 1.00 (Reference) | 1.00 (Reference) |
| DPP-4 inhibitors | 661,669 | 11 | 1,338,139 | 0.82 | 2.33 (0.93–5.88) | 2.52 (0.98–6.45) |

DPP-4, dipeptidyl peptidase-4; non-DPP4i-DMRx, non-DPP-4 inhibitor diabetes medications; HR, hazard ratio; CI, confidence interval. * Testing global null hypothesis, p-value 0.0691.

**Supplementary Table S5.** Subgroup analysis of the association between DPP-4 inhibitor use and the risk of pemphigus vulgaris (PV) using a time-varying analysis

| Exposure | Crude HR (95% CI) | Adjusted HR (95% CI) |
| --- | --- | --- |
| Age |  |  |
| Age <65 |  |  |
| Non-DPP4i-DMRx | 1.00 (Reference) | 1.00 (Reference) |
| DPP-4 inhibitors | 3.09 (1.01–9.48) | 3.15 (1.01–9.81) |
| Age ≥65 |  |  |
| Non-DPP4i-DMRx | 1.00 (Reference) | 1.00 (Reference) |
| DPP-4 inhibitors | 3.09 (1.01–9.48) | 2.29 (0.32–16.35) |
| Sex |  |  |
| Female |  |  |
| Non-DPP4i-DMRx | 1.00 (Reference) | 1.00 (Reference) |
| DPP-4 inhibitors | 3.09 (1.01–9.48) | 3.22 (1.03–10.04) |
| Male |  |  |
| Non-DPP4i-DMRx | 1.00 (Reference) | 1.00 (Reference) |
| DPP-4 inhibitors | 3.09 (1.01–9.48) | 13.74 (2.08–90.65) |
| BMI, kg/m^2^ |  |  |
| <18.5 (Underweight) |  |  |
| Non-DPP4i-DMRx | 1.00 (Reference) | 1.00 (Reference) |
| DPP-4 inhibitors | 3.09 (1.01–9.48) | NA |
| 18.5~23 (Normal weight) |  |  |
| Non-DPP4i-DMRx | 1.00 (Reference) | 1.00 (Reference) |
| DPP-4 inhibitors | 3.09 (1.01–9.48) | 3.22 (1.03–10.06) |
| >23 (Overweight) |  |  |
| Non-DPP4i-DMRx | 1.00 (Reference) | 1.00 (Reference) |
| DPP-4 inhibitors | 3.09 (1.01–9.48) | 7.21 (0.69–75.67) |

NA, not applicable; DPP-4, dipeptidyl peptidase-4; non-DPP4i-DMRx, non-DPP-4 inhibitor diabetes medications; HR, hazard ratio; 95% CI, 95% confidence interval.

**Supplementary Table S6.** Analysis of the association between the use of individual DPP-4 inhibitors and the risk of pemphigus foliaceus (PF) using time-varying analysis

| Exposure |  |  |  |  |  |
| --- | --- | --- | --- | --- | --- |
| Individual drugs | Events  (PF) | Person-years | Incidence rate (per 100,000 person-years) | Crude HR (95% CI) | Adjusted HR (95% CI) |
| Non-DPP4i-DMrx | 7 | 1,092,603 | 0.64 | 1.00 (Reference) | 1.00 (Reference) |
| Sitagliptin | 1 | 215,637 | 0.46 | 0.89 (0.11–7.26) | 0.98 (0.12–8.29) |
| Non-DPP4i-DMrx | 7 | 1,092,732 | 0.64 | 1.00 (Reference) | 1.00 (Reference) |
| Vildagliptin | 0 | 60,879 | 0 | NA | NA |
| Non-DPP4i-DMrx | 7 | 1,092,769 | 0.64 | 1.00 (Reference) | 1.00 (Reference) |
| Saxagliptin | 0 | 35,120 | 0 | NA | NA |
| Non-DPP4i-DMrx | 7 | 1,092,550 | 0.64 | 1.00 (Reference) | 1.00 (Reference) |
| Linagliptin | 1 | 168,209 | 0.59 | 1.16 (0.14–9.42) | 1.48 (0.17–12.65) |
| Non-DPP4i-DMrx | 7 | 1,092,646 | 0.64 | 1.00 (Reference) | 1.00 (Reference) |
| Gemigliptin | 0 | 114,660 | 0 | NA | NA |
| Non-DPP4i-DMrx | 7 | 1,092,723 | 0.64 | 1.00 (Reference) | 1.00 (Reference) |
| Alogliptin | 0 | 35,128 | 0 | NA | NA |
| Non-DPP4i-DMrx | 7 | 1,092,713 | 0.64 | 1.00 (Reference) | 1.00 (Reference) |
| Teneligliptin | 1 | 42,903 | 2.33 | 3.83 (0.47–31.20) | 5.37 (0.63–45.42) |
| Non-DPP4i-DMrx | 7 | 1,092,764 | 0.64 | 1.00 (Reference) | 1.00 (Reference) |
| Anagliptin | 0 | 13,151 | 0 | NA | NA |
| Non-DPP4i-DMrx | 7 | 1,092,742 | 0.64 | 1.00 (Reference) | 1.00 (Reference) |
| Evogliptin | 0 | 19,977 | 0 | NA | NA |

NA, not applicable; DPP-4, dipeptidyl peptidase-4; non-DPP4i-DMRx, non-DPP-4 inhibitor diabetes medications; HR, hazard ratio; CI, confidence interval.

**Supplementary Table S7.** Analysis of the association between DPP-4 inhibitor use and the risk of pemphigus foliaceus (PF) using time-varying analysis over different extension periods, including the 60-day, 90-day, and 180-day extension periods.

| Exposure | Patients | Events  (PF) | Person-years | Incidence rate (per 100,000 person-years | Crude HR (95% CI) | Adjusted HR (95% CI) |
| --- | --- | --- | --- | --- | --- | --- |
| 60-day extension period | |  |  |  |  |  |
| Non-DPP4i-DMRx | 900,852 | 9 | 1,311,434 | 0.69 | 1.00 (Reference) | 1.00 (Reference) |
| DPP-4 inhibitors | 613,919 | 4 | 1,115,010 | 0.36 | 0.61 (0.19–1.99) | 0.66 (0.20–2.21) |
| 90-day extension period | |  |  |  |  |  |
| Non-DPP4i-DMRx | 900,873 | 9 | 1,407,898 | 0.64 | 1.00 (Reference) | 1.00 (Reference) |
| DPP-4 inhibitors | 633,367 | 4 | 1,213,390 | 0.33 | 0.61 (0.19–1.99) | 0.66 (0.20–2.22) |
| 180-day extension period | |  |  |  |  |  |
| Non-DPP4i-DMRx | 900,888 | 9 | 1,532,095 | 0.59 | 1.00 (Reference) | 1.00 (Reference) |
| DPP-4 inhibitors | 661,669 | 5 | 1,338,150 | 0.37 | 0.76 (0.25–2.30) | 0.84 (0.27–2.58) |

DPP-4, dipeptidyl peptidase-4; non-DPP4i-DMRx, non-DPP-4 inhibitor diabetes medications; HR, hazard ratio; 95% CI, 95% confidence interval.

**Supplementary Table S8.** Subgroup analysis of the association between DPP-4 inhibitor use and pemphigus foliaceus (PF) risk using a time-varying analysis

| Exposure |  |  |
| --- | --- | --- |
|  | Crude HR (95% CI) | Adjusted HR (95% CI) |
| Age |  |  |
| Age <65 |  |  |
| Non-DPP4i-DMRx | 1.00 (Reference) | 1.00 (Reference) |
| DPP-4 inhibitors | 0.84 (0.24–2.88) | 0.87 (0.25–3.07) |
| Age ≥65 |  |  |
| Non-DPP4i-DMRx | 1.00 (Reference) | 1.00 (Reference) |
| DPP-4 inhibitors | 0.84 (0.24–2.88) | 1.24 (0.19–8.20) |
| Sex |  |  |
| Female |  |  |
| Non-DPP4i-DMRx | 1.00 (Reference) | 1.00 (Reference) |
| DPP-4 inhibitors | 0.84 (0.24–2.88) | 0.89 (0.25–3.14) |
| Male |  |  |
| Non-DPP4i-DMRx | 1.00 (Reference) | 1.00 (Reference) |
| DPP-4 inhibitors | 0.84 (0.24–2.88) | 1.22 (0.19–7.77) |
| BMI, kg/m^2^ |  |  |
| <18.5 (Underweight) |  |  |
| Non-DPP4i-DMRx | 1.00 (Reference) | 1.00 (Reference) |
| DPP-4 inhibitors | 0.84 (0.24–2.88) | NA |
| 18.5~23 (Normal weight) |  |  |
| Non-DPP4i-DMRx | 1.00 (Reference) | 1.00 (Reference) |
| DPP-4 inhibitors | 0.84 (0.24–2.88) | 0.88 (0.25–3.12) |
| >23 (Overweight) |  |  |
| Non-DPP4i-DMRx | 1.00 (Reference) | 1.00 (Reference) |
| DPP-4 inhibitors | 0.84 (0.24–2.88) | 0.43 (0.06–3.10) |

NA, not applicable; DPP-4, dipeptidyl peptidase-4; non-DPP4i-DMRx, non-DPP-4 inhibitor diabetes medications; HR, hazard ratio; 95% CI, 95% confidence interval.

**Supplementary Table S9.** The specification of the target trial and its emulation.

| Component | Target trial | Emulated trial using real-world data |
| --- | --- | --- |
| Design | Open-label two-parallel arm randomised trial |  |
| Aim | Determine whether type 2 diabetes patients using DPP-4 inhibitors have an increased incidence of PV or PF compared to type 2 diabetes patients using non-DPP-4 inhibitor diabetes medications (non-DPP4i-DMRx) | Same |
| Eligibility | Participants aged 30 or older, newly diagnosed with type 2 diabetes from 2010 to 2020, who were prescribed diabetes medication and underwent a health screening exam within two years before their type 2 diabetes diagnosis | Same |
| Exclusions | Patients with HIV, those previously diagnosed with type 1 diabetes before cohort enrolment, initial treatment of type 2 diabetes with insulin, and presence of autoimmune bullous disease prior to enrolling in the cohort | Same |
| Treatment strategies | 1. DPP-4 inhibitors 2. Non-DPP4i-DMRx | Same |
| Treatment assignment | Patients are randomly assigned to either strategy | Patients are non-randomly assigned to a treatment strategy. Randomisation is emulated via cloning of patients in both arms. |
| Outcome | Incidence of PV (for PV risk analyses) or PF (for PF risk analyses) | Same |
| Type of outcome | Failure time | Same |
| Censoring | Loss to follow up | Loss to follow up, administrative censoring |
| Adjustment variables | Age at first prescription of diabetes medication, sex, waist circumference, BMI, fasting blood glucose, systolic/diastolic blood pressure, and pre-existing comorbidities | Same |
| Causal contrast | Intention-to-treat effect; per-protocol effect | Per protocol: In each arm of the emulated trial, patients who deviate from the protocol are censored at their time of deviation |
| Estimands | Hazard ratio between arms | Same |

**Supplementary Table S10.** Distributions of inverse probability of treatment weights and effective sample sizes before and after Winsorization in the pemphigus vulgaris (PV) analysis

|  | Before Winsorization | | |  | After Winsorization | | | | | |
| --- | --- | --- | --- | --- | --- | --- | --- | --- | --- | --- |
| Characteristics | DPP-4 inhibitors used  N = 571,304 | Non-DPP4i-DMRx  N = 653,438 |  | | |  | DPP-4 inhibitors used  N = 571,304 | Non-DPP4i-DMRx  N = 653,438 |  |  |
|  |  |  |  |  |  |  |  |  |  |  |
| Mean weight (SD) | 2.13 (0.43) | 1.91 (2.31) |  | | |  | 2.13(0.43) | 1.86(0.43) |  |  |
| Median | 2.19 | 1.74 |  | | |  | 2.19 | 1.74 |  |  |
| Range of weight | 1.001–4.1 | 1.27–1,126.57 |  | | |  | 1.27–3.77 | 1.27–3.77 |  |  |
| ESS | 548,932 | 326,719 |  | | |  | 548,932 | 620,314 |  |  |
| ESS% | 96.1% | 50.0% |  | | |  | 96.1% | 94.9% |  |  |

SD, standard deviation. ESS, effective sample size.

**Supplementary Appendix 1.** Details on the data sources, study design, and patient selection

A retrospective cohort study was conducted using the Korean National Health Insurance (NHI) database. Patients with type 2 diabetes who were identified by ICD-10-CM codes between January 1, 2002, and December 31, 2020, were included in the study. To assemble a newly diagnosed cohort, we excluded individuals with prior diabetes diagnoses from January 1, 2002, through December 31, 2009. New cases were defined as those receiving a first-ever prescription for an antidiabetic medication following diagnosis. Patients who met the specified criteria were considered eligible if they were over the age of 30 years at the time of their initial prescription and had undergone a National Health Screening Program (NHSP) examination within two years prior to diagnosis. In the event that multiple NHSP records were available, the assessment that was most proximate to the diagnosis was utilized. We excluded patients with HIV due to its known association with bullous disease, those with a history of autoimmune bullous disorders before enrollment, those with type 1 diabetes, and those who initiated insulin as first-line therapy for type 2 diabetes, as they represent a group with different disease severity.

**Supplementary Appendix 2.** Details on the statistical analysis

We applied five statistical approaches to evaluate the association between DPP-4 inhibitor use and pemphigus. Model 1 was an extended Cox proportional hazards model in which diabetes medication use was included as a time-varying covariate to address immortal time bias. Model 2 used the clone-censor-weight method. This approach emulates the methodology of a clinical trial, whereby two copies of all patients are created at the time of cohort entry, with one copy assigned to the treatment group and the other to the control group [1, 2]. Each clone was censored on deviation from the protocol of the allocated group. The treatment group is required to receive treatment within a specified timeframe, which is referred to as the "grace period." If treatment is not received within this designated period, it is censored. In the control group, patients were censored if they received treatment within the grace period. In this study, the grace period between the cohort enrollment and the initiation of treatment with a DPP-4 inhibitor was set 2 years. The 2-year grace period was selected to allow sufficient time for participants to initiate DPP-4 inhibitor treatment. The application of the clone-censor-weight approach (Model 2) enabled the elimination of baseline confounding in patients with type 2 diabetes, as all patients were assigned to the DPP-4 inhibitor and non-DPP4i-DMrx groups at baseline. However, when protocol deviations occur after an assignment, informative censoring can result and lead to selection bias. To address this, inverse-probability-of-censoring weights (IPCW) were used. IPCW were estimated from a Cox proportional hazards model that included age at initial diabetes medication prescription, sex, comorbidities (psoriasis, dementia, Parkinson’s disease, epilepsy, and cancer), and National Health Screening Program measurements (body mass index, waist circumference, systolic/diastolic blood pressure, and fasting blood glucose) as covariates. Using these weights alone, we then fitted a weighted Cox proportional hazards model—without additional covariate adjustments—to estimate the hazard ratio for the DPP-4 inhibitor effect. Model 3 was based on the structure of Model 1 but was modified to additionally consider patients who initiated DPP-4 inhibitors and subsequently switched to non–DPP4i-DMRx. Model 4 further extended this framework by allowing multiple treatment switches to capture real-world on–off treatment patterns. Model 5 was an alternative analytic approach that used inverse-probability-of-treatment weighting (IPTW) with stabilized weights to address measured confounding. In the IPTW analysis, a propensity score was calculated from a logistic regression model based on the covariates. After applying the IPTW, the covariate balance was evaluated through the utilization of standardized mean differences (SMDs). An absolute SMD of ≤ 0.25 was defined as adequate balance. A Cox proportional hazards model weighted by stabilized IPTW was fitted to assess the effect of DPP-4 inhibitors on outcomes. No additional covariate adjustment was performed beyond the application of stabilized IPTW. We also examined potential differences in the occurrence of pemphigus among individual DPP-4 inhibitors. For this analysis, we reconstructed separate study populations, each comprising users of a specific DPP-4 inhibitor compared with non-DPP-4 inhibitor users, using the Model 1. Subgroup analyses examined whether the association between DPP-4 inhibitor use and pemphigus risk varied by age (<65 vs. ≥65 years), sex (male vs. female), and body mass index category (underweight, normal weight, overweight), with corresponding interaction terms included in Cox models adjusted for all other covariates. We performed additional analyses based on the Model 1 by redefining the extension period from 30 to 60, 90, and 180 days. Models 1, 2, 3, and 4 were adjusted for the covariates described in the methods section. For models 3 and 4 with an extended Cox proportional hazards model, robust variance estimation was performed by the Lin–Wei robust variance estimator to account for patients who were included multiple times [3]. We reported hazard ratios (HRs) and their 95% confidence intervals (CIs). Statistical significance was defined as a two-sided P-value less than 0.05. Statistical analyses were performed using SAS Enterprise Guide 7.15 (SAS Institute, Cary, NC, USA).

**References**

1	Petito LC, García-Albéniz X, Logan RW et al. Estimates of Overall Survival in Patients With Cancer Receiving Different Treatment Regimens: Emulating Hypothetical Target Trials in the Surveillance, Epidemiology, and End Results (SEER)-Medicare Linked Database. *JAMA Netw Open* 2020; **3**(3): e200452. DOI: 10.1001/jamanetworkopen.2020.0452.

2	Duchesneau ED, Jackson BE, Webster-Clark M et al. The Timing, the Treatment, the Question: Comparison of Epidemiologic Approaches to Minimize Immortal Time Bias in Real-World Data Using a Surgical Oncology Example. *Cancer Epidemiol Biomarkers Prev* 2022; **31**(11): 2079-2086. DOI: 10.1158/1055-9965.Epi-22-0495.

3	Wei LJ, Y. LD, and Weissfeld L. Regression Analysis of Multivariate Incomplete Failure Time Data by Modeling Marginal Distributions. *Journal of the American Statistical Association* 1989; **84**(408): 1065-1073. DOI: 10.1080/01621459.1989.10478873.
